# Prognostic factors and outcomes of CT-guided Iodine-125 seed brachytherapy for retroperitoneal lymph node metastases from a previously irradiated field in cervical carcinoma: a multicenter retrospective study

**DOI:** 10.3389/fonc.2026.1897353

**Published:** 2026-07-22

**Authors:** Shule Ren, Xuemin Di, Shaojun Zhao, Zhen Gao, Huimin Yu, Jinxin Zhao, Chuande Zheng, Shifeng Liu, Wukui Huang, Hongtao Zhang

**Affiliations:** 1 Department of Oncology, Wardne, Hebei General Hospital, Shijiazhuang, China; 2 Graduate School Hebei Medical University, Shijiazhuang, China; 3 Department of Interventional Diagnosis and Treatment, Affiliated Cancer Hospital of Xinjiang Medical University, Urumqi, China; 4 Qingdao University Affiliated Hospital Interventional Medicine Center, Qingdao, China

**Keywords:** brachytherapy, cervical carcinoma, iodine-125 seed, multicenter research, retroperitoneal lymph node metastasis

## Abstract

**Objective:**

This retrospective study aimed to evaluate the efficacy, safety, and prognostic factors of Iodine-125 seed brachytherapy for Retroperitoneal Lymph Node Metastases (RLNM) from a Previously Irradiated Field in Cervical Carcinoma.

**Methods:**

Between March 2020 and November 2024, 33 patients (40 lesions) with RLNM from Cervical Carcinoma, who were ineligible for or declined further external beam radiotherapy (EBRT), underwent CT-guided iodine-125 seed brachytherapy at three tertiary hospitals. The follow-up deadline was 28/02/2026. The following endpoints were assessed: disease control rate (DCR), local tumor progression-free survival (LTPFS), overall survival (OS), and adverse events.

**Results:**

The median follow-up time was 18.6 months (range, 2.0-38.3 months), and the 3- and 6- month DCR was 90.0% and 77.5%, respectively. The median LTPFS was 9.0 months (IQR, 5.3 - 20.5), and the median OS was 20.0 months (IQR, 8.0 - 25.0). The 6-, 12-, 18- and 24- month survival rates were 81.8%, 60.5%, 50.2%, 28.2%, respectively. Among 8 patients with preoperative pain, the Numerical Rating Scale (NRS) score decreased from 3.50 ± 1.77 to 1.75 ± 1.49 one month postoperatively (t=3.564, P = 0.009), a statistically significant difference. One patient developed a sigmoid colon fistula 4 months postoperatively. Four patients experienced postoperative low back discomfort with NRS scores of 1-2, which was relieved spontaneously or with oral analgesics. Multivariate analysis using the Cox proportional hazards model showed that a dose to 90% of the gross tumor volume (D90) ≥ 108 Gy was a protective factor for LTPFS, and tumor invasion of the intestinal wall was a risk factor for OS.

**Conclusion:**

Iodine-125 seed brachytherapy is safe and effective for treating RLNM in Cervical Carcinoma. A D90 ≥ 108 Gy results in better LTPFS. Tumor invasion of the intestinal wall is a risk factor for OS.

## Introduction

Cervical carcinoma ranks as the fourth most common malignancy in women worldwide in terms of both incidence and mortality. Its incidence has continued to exhibit an upward trend (1). Concurrent chemoradiotherapy, with or without immunotherapy, constitutes the standard first treatment regimen for patients with locally advanced cervical cancer. Nonetheless, a proportion of patients develop disease recurrence after treatment, with studies reporting a median time to recurrence of 15.5 months following therapy. The 1-year and 5-year survival rates for patients with recurrent disease are 90.6% and 54.0%, respectively (2). This malignant tumor can metastasize through the lymphatic system. In 2018, the International Federation of Gynecology and Obstetrics (FIGO) classified lymph node metastasis (LNM) as an independent stage IIIC. Metastatic lesions can induce clinical manifestations such as abdominal pain, radiating back pain, and abdominal distension, and are correlated with a poor prognosis (3–6). Cho et al. reported that the 3-year survival rate for cervical cancer patients with para-aortic lymph node metastasis was only 42.7%, while the rate for those with distant metastasis was merely 7.7% (7).

Systemic therapy with or without radiotherapy constitutes the mainstay of treatment for recurrent or advanced disease, whereas local treatment should be prioritized for cervical cancer patients with low rates of recurrence or metastasis (8). Primary local treatment modalities encompass surgery and radiotherapy. Surgical resection frequently fails to achieve radical eradication of lesions, and is associated with high morbidity and mortality due to complications such as vesicovaginal fistula, rectovaginal fistula, and the occurrence of three or more complications (9, 10), thereby rendering it clinically impractical for widespread implementation. Radiotherapy is a crucial local treatment modality. However, for patients who have previously undergone radiotherapy, the cumulative radiation dose to organs at risk (OARs) presents a challenge that cannot be overlooked. Currently, re-irradiation options primarily include stereotactic body radiation therapy (SBRT) and brachytherapy (11). While SBRT demonstrates acceptable efficacy for treating abdominopelvic lymph node metastases (12–14), its application in the re-irradiation setting is associated with a higher risk of radiation-induced toxicity (15). Brachytherapy can be further categorized into intracavitary brachytherapy and iodine-125 seed brachytherapy. Compared to intracavitary brachytherapy, iodine-125 seed brachytherapy is better suited for treating non-central recurrences (11), offering advantages such as superior conformality and shorter procedure times. Furthermore, studies have indicated that compared to SBRT, seed implantation can deliver comparable doses to the planning target volume (PTV) while exposing OARs to significantly lower doses (16). A consensus statement proposes that iodine-125 seed brachytherapy is a viable option for patients with oligometastatic or symptomatic invasive extra-pelvic recurrences who are not suitable candidates for external beam radiotherapy (17).

Existing research has confirmed the safety and efficacy of iodine-125 seed brachytherapy for treating RLNM from Cervical Carcinoma (18). However, high-level evidence regarding optimal dosimetric parameters (e.g., prescription dose, D90) and their impact on local control and survival remains scarce. This multicenter study aimed to evaluate the efficacy and safety of iodine-125 seed brachytherapy for RLNM from Cervical Carcinoma and, more importantly, to identify independent prognostic factors, particularly dosimetric and anatomical ones, to guide clinical practice.

## Method

The follow-up deadline was 28/02/2026. All patients signed informed consent for radioactive seed brachytherapy. This retrospective study was approved by the Ethics Committee of the Affiliated Cancer Hospital of Xinjiang Medical University (No. K-2025102), the Medical Ethics Committee of Hebei General Hospital (No. 2026-LW-117), and the Medical Ethics Committee of the Affiliated Hospital of Qingdao University (No. QYFYWZLL50026).From March 2020 to November 2024, 33 patients with RLNM from Cervical Carcinoma were selected from three medical institutions (Department of Oncology Ward 1, Hebei General Hospital; Department of Interventional Diagnosis and Treatment, Affiliated Tumor Hospital of Xinjiang Medical University; and Interventional Medicine Center, Affiliated Hospital of Qingdao University), totaling 40 lesions. Imaging or endoscopic examinations indicated that 20 lesions invaded the ureter and 12 invaded the intestinal wall.

### Inclusion and exclusion criteria

Inclusion criteria: (1) Pathologically confirmed Cervical Carcinoma via surgery or hysteroscopic biopsy; patients refused or were unsuitable for surgery, EBRT, etc. (2) Patients with a controllable primary tumor who experience recurrence after RLNM radiotherapy and who refuse or are not suitable for surgery or external beam radiotherapy. Exclusion criteria: (1) Cardiopulmonary or other organ failure or severe coagulation dysfunction uncorrectable after standard treatment. (2) Receiving Iodine-125 seed brachytherapy combined with other local therapies such as thermal ablation or EBRT. (3) Residual tissue remains in the primary sites such as the cervix and vagina.

### Iodine-125 seed brachytherapy

One week before implantation, patients underwent an abdominopelvic CT scan in the required surgical position (typically prone or supine, and in some cases, lateral). The imaging data were transferred to the treatment planning system (TPS) (Panther Brachy version 5.0 TPS; Prowess Inc., Concord, CA, USA). The gross tumor volume (GTV) and surrounding organs at risk (OARs) were delineated. the clinical target volume (CTV) was obtained by expanding the GTV by 5 mm in all directions. The OAR are mainly medulla spinalis, intestine, ureter, bladder, etc., The dose limit of endangering organs is referenced from the dose parameters of endangering organs in the radioactive particle therapy of prostate cancer. Then the seed activity was 11.1 to 29.6MBq. the prescription dose was 80 to 140 Gy. The needle insertion paths, seed positions, and doses to the target volume and OARs were precisely designed, and a dose-volume histogram (DVH) was subsequently generated. Additionally, patients with ureteral obstruction underwent ureteral stent placement.

A radioactivity meter (RM−905a well−type ionization chamber, National Institute of Metrology, BeiJing, China) was used to measure the activity of radioactive 125I seeds (Model, 6711−99; Beijing ZHIBO Bio-Medical Technology Co., Ltd., China) before the operation. If activity error was < 5%, all seeds would be blocked in the shell of brachytherapy applicator for disinfection.

Needles were inserted, and seeds were implanted under guidance of ultrasound combined with CT. For patients with large tumors or those located in anatomically complex regions, seed implantation was guided by a 3D-printed template (Unicorn 3DSL450M; Beijing Unicorn Science and Technology Ltd., Beijing, China). Following needle removal, the puncture site was compressed for 10 to 20 minutes, after which an immediate CT scan was performed to assess for bleeding or hematoma. Upon safe return to the ward, sandbag compression was continued for 2 to 4 hours. Within one week post-implantation, an abdominopelvic CT scan was obtained, and the images were transferred to the treatment planning system (TPS) to evaluate seed distribution, perform postoperative dose verification, and conduct quality assessment.

### Follow-up and evaluation criteria

The primary endpoint was local tumor progression-free survival (LTPFS), defined as the time from Iodine-125 seed brachytherapy to local disease progression or death from any cause. The secondary endpoint was overall survival (OS), defined as the time from initiation of Iodine-125 seed brachytherapy to death from any cause. Local tumor response was assessed according to Response Evaluation Criteria in Solid Tumors (RECIST1.1) criteria, and the disease control rate (DCR) was calculated as the proportion of patients achieving complete response (CR), partial response (PR), or stable disease (SD). Pain severity was evaluated using the Numerical Rating Scale (NRS). Patients were closely monitored postoperatively and during follow-up for symptoms such as bleeding, hematoma, seed migration, and intestinal fistula. The severity of radiation-induced injuries was graded according to the Radiation Therapy Oncology Group/European Organization for Research and Treatment of Cancer criteria.

### Statistical analysis

The prescription dose for Iodine-125 Seed Brachytherapy was converted to an equivalent dose in 2-Gy fractions (EQD2). In a case where a daily fraction dose of d (Gy) was delivered in n fractions, EQD2 (Gy) was calculated as follows:

$EQD2=D\left[1+\frac{R_{0}}{\left(\mu +\lambda \right)\frac{\alpha }{\beta }}\right]/\left[1+\frac{2}{\frac{\alpha }{\beta }}\right],\;$ $R_{0}=D\times \lambda$, D is the D90 of brachytherapy, R_0_ is the initial dose rate, μ is the cell repair constant equals to 0.5h^-^¹. λ is the seed decay constant equals to ln2/T_1_/_2_, α/β ratio of 10 was used to obtain the EQD2 for the tumor. Statistical analysis was performed using SPSS 25.0 software. Quantitative data are described as mean ± standard deviation (mean ± SD) or median (interquartile range) [M (P25, P75)]. Qualitative data are expressed as frequency or percentage. Comparisons of quantitative data were performed using the t-test or rank-sum test. Univariate and multivariate analyses were conducted using the Cox proportional hazards model. Normally distributed continuous variables were dichotomized into ≥ mean group and< mean group, while non−normally distributed continuous variables were categorized into ≥ median group and< median group. Variables with a P-value< 0.05 in the univariate analysis were subsequently entered into the multivariate Cox proportional hazards model using a forward stepwise selection method. Survival analysis was performed using the Kaplan-Meier method. P< 0.05 was considered statistically significant.

## Results

### Patient data

A total of 33 patients were included in the study. The median age was 53 years(IQR, 42 - 57). Patient characteristics are presented in **Table 1**. All treatment sites were partially included in a previous radiotherapy field. No intraoperative failures occurred. A total of 24 patients (72.7%) died. Among them, one patient’s cause of death was pulmonary embolism, one patient succumbed to myelodysplastic syndrome, and the remaining patients died due to disease progression.

**Table 1:** Patients’ characteristics.

Variable	Value/type/stage/size	No. of patients(n)	Percentage(%)
Age	<50	14	42.4
	≥50	19	57.6
Pathological type	Squamous cell carcinoma	30	90.9
	Adenocarcinoma	2	6.1
	Adenosquamous carcinoma	1	3.0
Diagnostic staging	Staging I	9	27.3
	Staging II	3	9.1
	Staging III	19	57.6
	Staging IV	2	6.0
Previous treatment	Surgery+Radiotherapy+Systemic therapy	26	78.8
	Radiotherapy+Systemic therapy	6	18.2
	Surgery+Radiotherapy	1	3.0
Distant metastasis	Yes	32	97.0
	No	1	3.0
Postoperative systemic therapy	Yes	28	84.8

**Figure 1:**
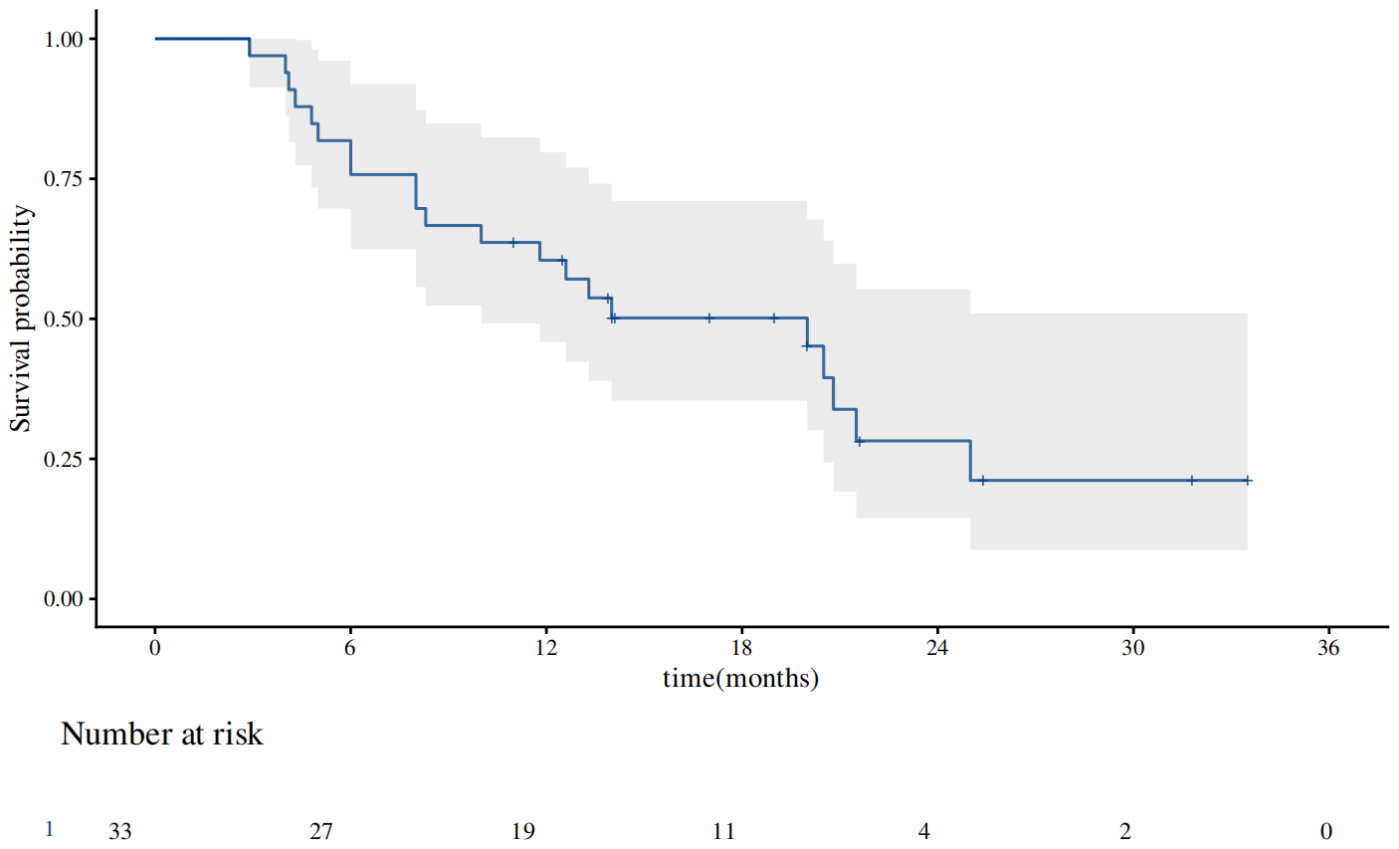
Survival analysis.

All patients successfully completed Iodine-125 seed brachytherapy. The DCR at 3 and 6 months postoperatively was 90.0% (36/40) and 77.5% (31/40), respectively. The median LTPFS was 9.0 months (IQR, 5.3 - 20.5; range, 1.3 - 38.3 months), and the median OS was 20.0 months (IQR, 8.0 - 25.0). The 6-, 12-, 18- and 24- month survival rates were 81.8%, 60.5%, 50.2%, 28.2%, respectively (survival analysis shown in **Figure 1**). 15 patients experienced target lesion recurrence. Among them, 6 patients showed no significant lesion enlargement during follow-up after repeat seed brachytherapy, 1 patient progressed despite repeat brachytherapy, and the remaining patients did not receive repeat seed brachytherapy. Among 8 patients with preoperative pain, the NRS score decreased from 3.50 ± 1.77 to 1.75 ± 1.49 one month postoperatively (t=3.564, P = 0.009), a statistically significant difference.

**Figure 2:**
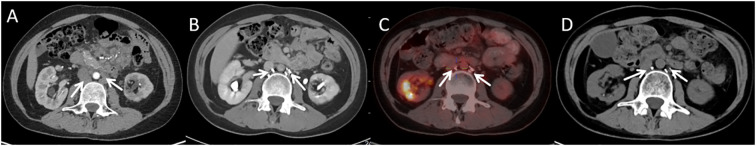
**(A)** Follow-up examinations revealed a retrocaval fused lymph node (approximately 15 mm × 13 mm) and a left para-aortic lymph node (approximately 32 mm × 18 mm); **(B)** Partial response was achieved one month post-implantation; **(C)** PET/CT scan at 7 months post-procedure showed no significant hypermetabolic activity within the target area; **(D)** Complete response was achieved at 14 months post-implantation.

A typical case is shown in **Figure 2**. A 43-year-old patient presented with initial FIGO stage IIIC1p disease. Previous treatments included surgery, radiotherapy (46.8 Gy/26 f), and a TP regimen combined with apatinib. Disease progression was detected on follow-up imaging at 8 months post-radiotherapy. The regimen was further modified to toripalimab plus apatinib following grade IV myelosuppression. Follow-up imaging identified a retrocaval fused lymph node (approximately 15 mm × 13 mm) and a left para-aortic lymph node (approximately 32 mm × 18 mm). The patient underwent radioactive seed brachytherapy. PR was achieved at 1 month post-procedure. PET-CT at 7 months post-treatment showed no significant hypermetabolic activity in the target region. The patient attained CR at 14 months post-implantation. OS was 20.5 months, and the patient died of progressive meningeal metastasis. The individual in this manuscript has given written informed consent to publish these case details.

### Adverse reactions

One patient with a lesion measuring 58mm×40mm in maximum cross-section, implanted with 50 seeds of 18.5 MBq activity and a postoperative D90 of 88 Gy, developed a sigmoid colon fistula 4 months postoperatively, constituting a grade 3 radiation toxicity reaction; the patient received only symptomatic treatment such as anti-infection. Four patients experienced postoperative low back discomfort with NRS scores of 1-2, relieved spontaneously or with oral analgesics. No significant intraoperative bleeding or local hematoma occurred. No other adverse reactions related to seed brachytherapy, such as radiation proctitis, radiation cystitis, or needle tract metastasis, were observed.

### Clinical efficacy analysis

**Figure 3:**
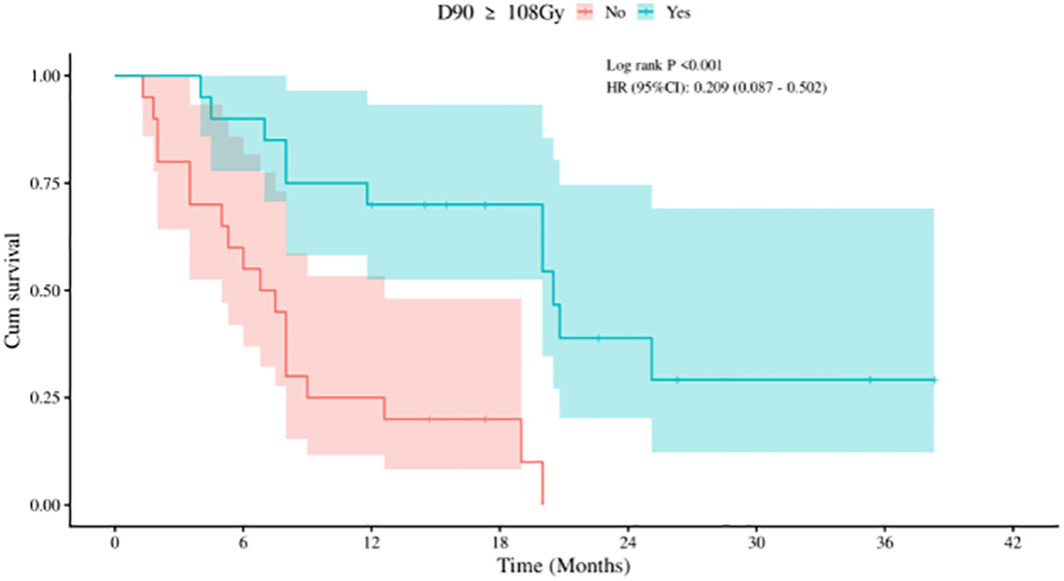
Local tumor progression-free survival in patients with D90≥108 Gy and D90<108 Gy.

Univariate Cox regression analysis of factors including diagnostic staging, maximum diameter, D90, Postoperative systemic therapy, ureteral invasion, and intestinal wall invasion with LTPFS showed that D90, ureteral invasion, and intestinal wall invasion were influencing factors for LTPFS. Variables with P<0.05 were included in multivariate analysis, which showed that D90 ≥108 Gy was a protective factor for LTPFS (HR = 0.209, 95%CI 0.087 - 0.502, P<0.001) (**Table 2**). Patients who achieved a postoperative D90 ≥ 108 Gy had significantly longer LTPFS compared to those with D90< 108 Gy (Log-rank P<0.001) (**Figure 3**).

**Table 2:** Univariate and multivariate cox proportional hazards regression analysis of LTPFS for retroperitoneal lymph node metastases from cervical carcinoma.

	Univariate analysis		Multivariate analysis	
	HR (95% CI)	P-value	HR (95% CI)	P-value
Diagnostic staging (I+II vs. III+IV)*	0.873 (0.412 - 1.851)	0.723		
maximum diameter (≥31mm vs. <31mm)	1.767 (0.841 - 3.715)	0.133		
D90(≥108Gy vs. <108Gy)	0.209 (0.087 - 0.502)	<.001	0.209 (0.087 - 0.502)	<.001
Postoperative systemic therapy (Yes vs. No)	0.597 (0.207 - 1.721)	0.340		
involvement of the ureter (Yes vs. No)	4.034 (1.727 - 9.422)	0.001		
intestinal wall invasion (Yes vs. No)	4.249 (1.931 - 9.352)	<.001		

*Comparison between combined stages I–II and stages III–IV.

**Figure 4:**
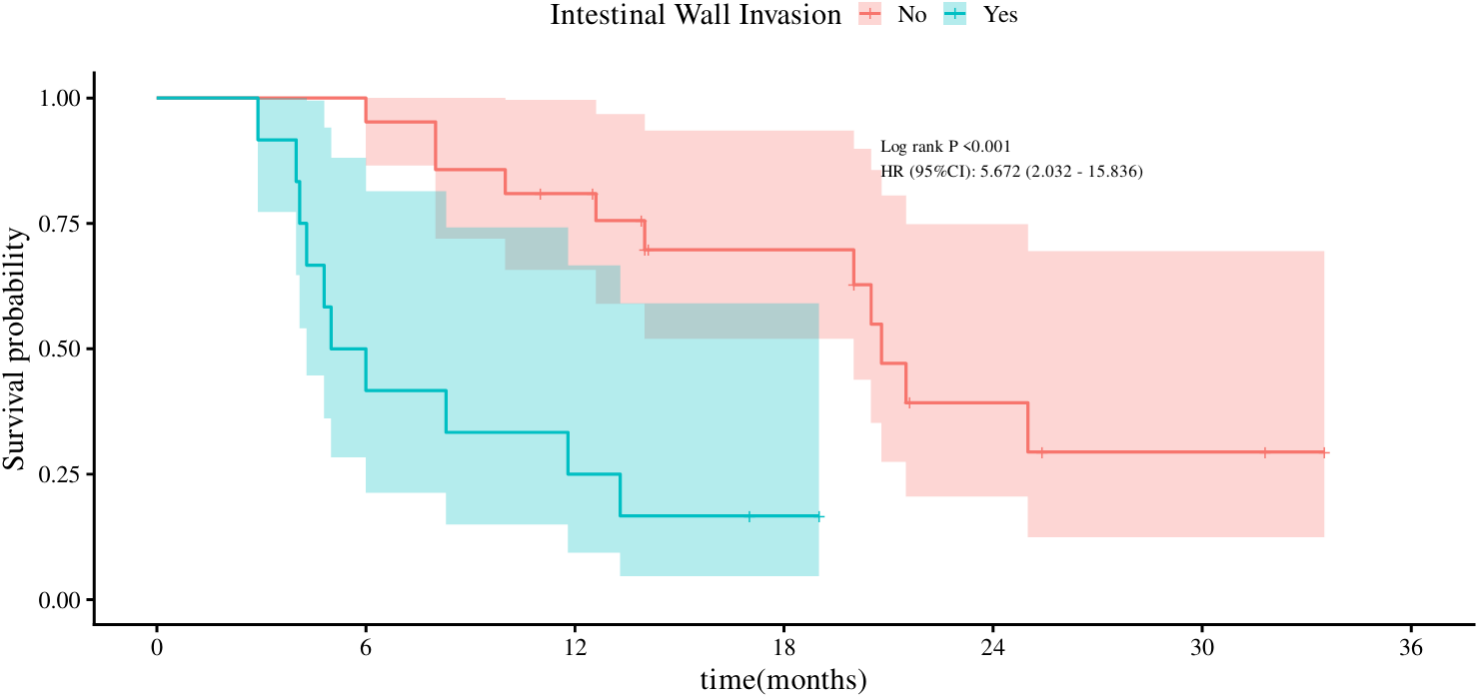
Overall survival between patients with and without intestinal wall invasion.

Univariate Cox analysis of the above factors with OS showed that ureteral invasion, and intestinal wall invasion were influencing factors for OS. Variables with P<0.05 were included in multivariate analysis, which showed that intestinal wall invasion was an independent risk factor for OS (HR = 5.672, 95%CI 2.032 ~ 15.836, P<0.001) (**Table 3**). Patients without intestinal wall invasion had significantly higher OS than those with intestinal wall invasion (Log−rank P<0.001) (**Figure 4**).

**Table 3:** Univariate and multivariate cox proportional hazards regression analysis of OS for retroperitoneal lymph node metastases from cervical carcinoma.

	Univariate analysis		Multivariate analysis	
	HR (95% CI)	P-value	HR (95% CI)	P-value
Diagnostic staging (I+II vs. III+IV)*	0.645 (0.273 ~ 1.523)	0.317		
Postoperative systemic therapy (Yes vs. No)	0.386 (0.087 ~ 1.715)	0.211		
involvement of the ureter (Yes vs. No)	4.638 (1.661 ~ 12.947)	0.003		
intestinal wall invasion (Yes vs. No)	5.672 (2.032 ~ 15.836)	<.001	5.672 (2.032 ~ 15.836)	<.001

*Comparison between combined stages I–II and stages III–IV.

## Discussion

Management of RLNM in cervical carcinoma remains a significant challenge, particularly for patients who have exhausted external beam radiotherapy options. In this context, Iodine-125 seed brachytherapy offers a valuable salvage modality.

Iodine-125 seed brachytherapy demonstrates effectiveness in managing recurrent/metastatic Cervical Carcinoma. In our investigation, The median LTPFS was 9.0 months (IQR, 5.3 - 20.5), and the median OS was 20.0 months (IQR, 8.0 - 25.0). The 6-, 12-, 18- and 24- month survival rates were 81.8%, 60.5%, 50.2%, 28.2%, respectively. These LTPFS and OS outcomes are consistent with outcomes reported in prior studies of brachytherapy for recurrent pelvic and retroperitoneal disease in Cervical Carcinoma (18–21). eight patients presenting with pain exhibited a marked reduction in NRS scores at one month post-implantation, aligning with previously published findings (22–24). These results should be interpreted with caution and are primarily indicative of the palliative potential of the procedure.

In this series, one patient developed a sigmoid colon fistula four months post-procedure. The target lesion in this case exhibited a maximum cross-section of approximately 58 mm × 40 mm, and preoperative imaging suggested invasion of the intestinal wall. Postoperative dosimetric verification revealed a D90 of 88 Gy for the target lesion, Several mechanisms may underlie fistula formation: (1) During post-implantation tumor necrosis, inadequate absorption of necrotic debris and insufficient fibrous tissue repair could predispose to intestinal wall injury. (2) Inhomogeneous dose distribution, potentially resulting in localized overdosage within areas of intestinal wall infiltration, may exacerbate radiation-induced intestinal damage. (3) The large lesion size, accompanied by liquefactive necrosis, could compromise the accuracy of brachytherapy and dose homogeneity. (4) Inherent tumor progression may also be a contributing factor. the case underscores the critical importance of risk stratification and dosimetric optimization, which are central themes of our findings.

Dosimetry constitutes a critical determinant of efficacy in Iodine-125 seed brachytherapy. Domestic consensus guidelines recommend a prescription dose range of 110–160 Gy for Iodine-125 seed brachytherapy targeting retroperitoneal metastases from Cervical Carcinoma (17). However, there is no established recommended dose for iodine-125 seed brachytherapy in the treatment of non-central recurrences occurring within a previously irradiated field in cervical carcinoma. Our study identified D90 as a significant prognostic factor for LTPFS, with a threshold of D90 ≥ 108 Gy associated with superior outcomes. This finding is concordant with reports by Di and Luo Aihua (20, 23, 25), who observed enhanced efficacy with D90 values ≥108.5-127.5 Gy in Cervical Carcinoma brachytherapy. In this series, The median EQD2 was 90.9 Gy (IQR, 78.0 - 104.4), which is higher than the 64.0 Gy (range, 31.3 - 95.1 Gy) reported in some re-irradiation series (26) and may contribute to the encouraging early survival rates observed. whereas the rapid decline in survival rate is likely associated with poor prognosis due to distant metastasis and organ invasion.

Furthermore, our analysis identified intestinal wall invasion as an independent adverse prognostic factor for OS. Potential explanations include: firstly, patients with intestinal wall invasion frequently present with concurrent systemic metastases, indicative of more aggressive tumor biology and an inherently poorer prognosis; secondly, the indistinct interface between the tumor and intestinal wall complicates accurate OAR and target volume delineation; thirdly, lesions adjacent to the bowel are prone to dosimetric cold and hot spots, potentially compromising treatment efficacy. The single fistula case underscores the stakes. In contrast, the other 11 patients with imaging evidence of intestinal wall invasion did not develop this complication. A review of their treatment planning revealed that in these cases, the prescribed dose near the intestinal wall was intentionally reduced in the preoperative plan (typically maintained at 60–80 Gy), and seeds with lower activity (e.g., 11.1-18.5 MBq) were utilized for placement in proximity to the intestinal wall. Furthermore, rigorous postoperative dose verification confirmed that the radiation dose delivered to the intestinal wall remained within tolerance limits (D2cc< 100% of the prescription dose, D0.1cc< 200 Gy).Based on this analysis, we propose the following principles for Iodine-125 seed brachytherapy in patients with tumor invasion of the intestinal wall: (1) Meticulous preoperative assessment of the depth and extent of invasion, integrating endoscopic evaluation, magnetic resonance imaging (MRI), or positron emission tomography-computed tomography (PET-CT) as necessary to precisely delineate the relationship between the tumor and the intestinal wall.(2) Strict adherence to intestinal wall dose constraints during treatment planning, employing a “peripheral dose de-escalation” strategy utilizing low-activity seeds in areas of suspected invasion.(3) Intraoperative utilization of high-precision guidance techniques to avoid seed clustering or inadvertent seed placement within the intestinal wall.(4) Postoperative dosimetric verification, with particular attention to parameters such as D2cc and V100 for the intestinal wall.(5) For patients with extensive invasive disease and significant intestinal wall involvement, consideration of adjunctive measures such as intestinal stent placement or prophylactic ostomy to mitigate fistula risk. Future prospective studies are warranted to establish optimal dose constraints and surgical strategies tailored to this high-risk patient subset.

In contrast, ureteral invasion did not emerge as an independent risk factor for OS. This may be attributable to the implementation of a standardized, multidisciplinary management protocol: for patients with concurrent ureteral obstruction, preoperative ureteral stent placement was routinely performed. This intervention not only relieved the obstruction but also provided a clear radiographic marker for ureteral identification during the procedure, facilitating the maintenance of a safe distance between implanted seeds and the ureter, thereby achieving effective OAR sparing. This successful experience underscores the critical importance of thorough preoperative assessment and intervention in enhancing both the safety and efficacy of the procedure.

Systemic therapy with or without radiotherapy constitutes the mainstay of treatment for recurrent or advanced disease. Although Kubota H et al. demonstrated that concurrent chemoradiotherapy was associated with superior efficacy in the treatment of recurrent cervical cancer (27), the present study failed to detect a statistically significant difference associated with systemic therapy. The underlying reasons are mainly related to the study population, treatment context, and sample composition. The patients enrolled in this study were mostly cases of recurrent or metastatic disease following multiple lines of therapy. Some patients exhibited platinum resistance and received only targeted therapy or immunotherapy. The overall tumor burden was high, the prognosis was poor, and the treatment response rate was low, all of which made it difficult to demonstrate the benefit of systemic therapy. Furthermore, the limited sample size of the present study resulted in insufficient statistical power to effectively detect intergroup differences, The efficacy of radioactive seed implantation combined with systemic therapy warrants further investigation.

As a form of brachytherapy, Iodine-125 seed brachytherapy offers distinct advantages, including demonstrable efficacy, a low complication profile, and minimal invasiveness, providing a therapeutic option for patients with advanced Cervical Carcinoma. Nevertheless, further research is needed to refine the optimal prescription dose, establish definitive OAR tolerance doses, and determine the optimal integration of this local modality with systemic therapies specifically for Cervical Carcinoma patients with RLNM undergoing seed brachytherapy. The current study is limited by its relatively small sample size and retrospective design, which introduces the potential for selection bias. This study has several limitations. First, The retrospective, multicenter design of this study may be subject to selection bias, temporal bias, and heterogeneity in patient management. Second, although the sample size is meaningful for a brachytherapy investigation, it restricts the statistical power for subgroup analyses (e.g., only 5 lesions did not receive postoperative systemic therapy). Third, the dose prescription and EQD2 conversion relied on a uniform α/β ratio, which may not reflect individual variations in tumor and normal tissue radiobiology. Fourth, A mixed-effects model was not employed to analyze potential patient-level correlations due to the limited sample size. Furthermore, treating multiple lesions from the same patient as independent observations may violate the independence assumption, potentially affecting the precision of the LTPFS estimates. Future efforts should prioritize prospective studies with larger patient cohorts to validate these findings, thereby enhancing their scientific rigor and generalizability.

## Data Availability

The original contributions presented in the study are included in the article/supplementary material. Further inquiries can be directed to the corresponding author.

## References

[1] Bray F Laversanne M Sung H Ferlay J Siegel RL Soerjomataram I . Global cancer statistics 2022: GLOBOCAN estimates of incidence and mortality worldwide for 36 cancers in 185 countries. CA Cancer J Clin. (2024) 74:229–63. doi: 10.3322/caac.21834 38572751

[2] Li J Liu G Luo J Yan S Ye P Wang J . Cervical cancer prognosis and related risk factors for patients with cervical cancer: a long-term retrospective cohort study. Sci Rep. (2022) 12:13994. doi: 10.1038/s41598-022-17733-8 35978078 PMC9385852

[3] Hamada K Yamanoi K Hayashi N Kotani Y Matsumoto H Horikawa N . Re-evaluating prognostic factors for cervical cancer with lymph node metastasis: a Japanese multicenter cohort study based on FIGO 2018. Int J Clin Oncol. (2025) 30:584–92. doi: 10.1007/s10147-025-02697-2 39815053

[4] Aşıcıoğlu O Ateş S Varol F Yalta TD Sayın C . Oncologic outcome and recurrence patterns of clinical stage IB and IIA cervical cancer: A large retrospective analysis of a tertiary reference center. Int J Gynaecol Obstet. (2026) 172:295–304. doi: 10.1002/ijgo.70366 40662455 PMC12724023

[5] Singh AK Grigsby PW Rader JS Mutch DG Powell MA . Cervix carcinoma, concurrent chemoradiotherapy, and salvage of isolated paraaortic lymph node recurrence. Int J Radiat Oncol Biol Phys. (2005) 61:450–5. doi: 10.1016/j.ijrobp.2004.06.207 15667966

[6] Cibula D Dostálek L Jarkovsky J Mom CH Lopez A Falconer H . Post-recurrence survival in patients with cervical cancer. Gynecol Oncol. (2022) 164:362–9. doi: 10.1016/j.ygyno.2021.12.018 34955236 PMC9406127

[7] Cho WK Kim YI Park W Yang K Kim H Cha H . Para-aortic lymph node recurrence after curative radiotherapy for cervical cancer. Int J Gynecol Cancer. (2019) 29:1116–20. doi: 10.1136/ijgc-2019-000615 31474588

[8] Chinese Brachytherapy Society of China Anti-Cancer Association . Clinical guidelines of the diagnosis and therapy of recurrent and metastatic uterine cervical cancer[J. Chin J Radiat Oncol. (2025) 33:751–64. doi: 10.3760/cma.j.cn113030-20250428-00166 37127326

[9] Conte C Della Corte L Pelligra S Bifulco G Abate B Riemma G . Assessment of salvage surgery in persistent cervical cancer after definitive radio-chemotherapy: A systematic review. Medicina. (2023) 59:192. doi: 10.3390/medicina59020192 36837394 PMC9967015

[10] Van Kol K Ebisch R Piek J Zusterzeel P Vergeldt T Bekkers R . Salvage surgery for patients with residual disease after chemoradiation therapy for locally advanced cervical cancer: A systematic review on indication, complications, and survival. Acta Obstetricia Gynecologica Scandinavica. (2021) 100:1176–85. doi: 10.1111/aogs.14093 33469927 PMC8359234

[11] Shen Z Qu A Jiang P Jiang Y Sun H Wang J . Re-irradiation for recurrent cervical cancer: A state-of-the-art review. Curr Oncol. (2022) 29:5262–77. doi: 10.3390/curroncol29080418 35892987 PMC9331513

[12] von der Grün J Regnery S Hörner-Rieber J Hoegen-Saßmannshausen P Andratschke N Tanadini-Lang S . Multicenter analysis of MRI-guided, stereotactic body radiotherapy as ablative treatment for lymph nodal metastases of the abdomen and pelvis. Radiother Oncol. (2026) 214:111237. doi: 10.1016/j.radonc.2025.111237 41167279

[13] Onal C Gultekin M Oymak E Guler OC Yilmaz MT Yuce Sari S . Stereotactic radiotherapy in patients with oligometastatic or oligoprogressive gynecological Malignancies: a multi-institutional analysis. Int J Gynecol Cancer. (2020) 30:865–72. doi: 10.1136/ijgc-2019-001115 32273293

[14] Shenker R Stephens SJ Davidson B Chino J . Role of stereotactic body radiotherapy in gynecologic radiation oncology. Int J Gynecol Cancer. (2022) 32:372–9. doi: 10.1136/ijgc-2021-002466 35256426

[15] Van Werkhoven LA Cammareri E Hoogeman MS Nout RA Milder MTW Nuyttens JJME . Stereotactic body radiation therapy on abdominal-pelvic lymph node oligometastases: a systematic review on toxicity. Acta Oncol. (2024) 29:822–32. doi: 10.2340/1651-226X.2024.40681 39473177 PMC11541805

[16] Li R Zhang Y Yuan Y Lin Q Dai J Xu R . Dosimetric comparison of CT-guided iodine-125 seed stereotactic brachytherapy and stereotactic body radiation therapy in the treatment of NSCLC. PloS One. (2017) 12:e0187390. doi: 10.1371/journal.pone.0187390 29121047 PMC5679513

[17] Jiang P Zou L Wei L Cheng G Sun B Zhang F . Chinese expert consensus on iodine125 seed implantation for recurrent cervical cancer in 2021. Front Oncol. (2021) 11:700710. doi: 10.3389/fonc.2021.700710 34858802 PMC8630633

[18] Di X W Z Ren H . 125I seed implantation for the treatment of 10 cases of recurrent retroperitoneal lymph node metastasis after radiotherapy of cervical cancer. J Interventional Radiol. (2017) 26:137–41. doi: 10.3969/j.issn.1008-794X.2017.02.010

[19] Wang Y Ma Y Zou L Lei H Teng Y Ye F . Efficacy and safety of 125I seed implantation in the treatment of pelvic recurrent cervical cancer following radiotherapy: A single-arm meta-analysis of chinese patients. Cancer Rep (Hoboken). (2024) 7:e2147. doi: 10.1002/cnr2.2147 39158182 PMC11331501

[20] Di X Gao Z Yu H Liu X Zhao J Wang J . 125I seed brachytherapy for non-central pelvic recurrence of cervical cancer after external beam radiotherapy. Radiat Oncol. (2024) 19:70. doi: 10.1186/s13014-024-02454-1 38849839 PMC11162001

[21] Tong L Liu P Huo B Guo Z Ni H . CT-guided 125I interstitial brachytherapy for pelvic recurrent cervical carcinoma after radiotherapy. Onco Targets Ther. (2017) 17:4081–8. doi: 10.2147/OTT.S139571 28860816 PMC5566505

[22] Mai Q Mo Z He J Chen M Gou Q Shi F . Feasibility and clinical value of computed tomography-guided125I brachytherapy for pain palliation in patients with retroperitoneal lymph node metastases. J Cancer Res Ther. (2020) 16:372–8. doi: 10.4103/jcrt.JCRT_597_19 32474526

[23] Qu A Jiang P Sun H Jiang W Jiang Y Tian S . Efficacy and dosimetry analysis of image-guided radioactive 125I seed implantation as salvage treatment for pelvic recurrent cervical cancer after external beam radiotherapy. J Gynecologic Oncol. (2018) 30:e9. doi: 10.3802/jgo.2019.30.e9 30479093 PMC6304405

[24] Wang Z Lu J Gong J Zhang L Xu Y Song S . CT-guided radioactive ¹²^5^I seed implantation therapy of symptomatic retroperitoneal lymph node metastases. Cardiovasc Intervent Radiol. (2014) 37:125–31. doi: 10.1007/s00270-013-0613-3 23580117

[25] Luo A Yan Q Yang Z Li X . Short- and Long-Term Outcomes of Different Treatment Modalities for Pelvic RecurrentCervical Cancer After Radiotherapy: A Small-Sample Retrospective Study [J]. Chongqing Med J. (2026) 55(2):363–8. doi: 10.3969/j.issn.1671-8348.2026.02.021

[26] Ren X Fu Y Liu Z Lin X Qiu L Li Y . Image-guided interstitial brachytherapy for recurrent cervical cancer after radiotherapy: A single institution experience. Front Oncol. (2022) 12:943703. doi: 10.3389/fonc.2022.943703 35928866 PMC9344972

[27] Kubota H Tsujino K Sulaiman N Sekii S Matsumoto Y Ota Y . Comparison of salvage therapies for isolated para-aortic lymph node recurrence in patients with uterine cervical cancer after definitive treatment. Radiat Oncol (London England). (2019) 14:1442–6. doi: 10.1186/s13014-019-1442-6 31878944 PMC6933699

