# Supplementary material for: Prognostic factors and outcomes of CT-guided Iodine-125 seed brachytherapy for retroperitoneal lymph node metastases from a previously irradiated field in cervical carcinoma: a multicenter retrospective study
Source: Front Oncol. 2026 Jul 22;16:1897353. doi: 10.3389/fonc.2026.1897353 (PMC13437463; doi:10.3389/fonc.2026.1897353)
Supplement: Supplementary file 1 [file Table2.docx]

| Variables | Total (n = 33) | Postoperative systemic therapy (n = 28) | Without postoperative systemic therapy (n = 5) | Statistic | P |
| --- | --- | --- | --- | --- | --- |
|  |  |  |  |  |  |
| Age, Mean ± SD | 49.78 ± 11.22 | 50.70 ± 10.90 | 44.80 ± 12.87 | t=1.08 | 0.287 |
| involvement of the ureter, n(%) |  |  |  | - | 1.000 |
| No | 14 (42.42) | 12 (42.86) | 2 (40.00) |  |  |
| Yes | 19 (57.58) | 16 (57.14) | 3 (60.00) |  |  |
| Intestinal Wall Invasion, n(%) |  |  |  | - | 0.630 |
| No | 21 (63.64) | 17 (60.71) | 4 (80.00) |  |  |
| Yes | 12 (36.36) | 11 (39.29) | 1 (20.00) |  |  |
| Diagnostic staging, n(%) |  |  |  | - | 1.000 |
| Ⅰ+Ⅱ | 13 (39.39) | 11 (39.29) | 2 (40.00) |  |  |
| Ⅲ+Ⅳ | 20 (60.61) | 17 (60.71) | 3 (60.00) |  |  |
| t: t-test, -: Fisher exact | | | | | |
| SD: standard deviation | | | | | |

While we found no statistically significant differences in baseline characteristics between the two groups, the small sample size (n=5) in the non-systemic therapy group severely limits our ability to exclude selection bias. This comparison should be interpreted with caution.
